# A Very Good Joke Indeed!

**Published:** 1856-04

**Authors:** 


*A Very Good Joke Indeed!*—We find, in the "Peninsular Journal," for March, a page devoted to us. The opening paragraph contains the kernel of the nut, and we cut it out in order that our readers may appreciate the fun:

"*A Good Joke.*—The best one of the past month has been perpetrated by the gentlemanly editor of the Buffalo Medical Journal.

"After publishing in his own columns a communication signed 'R.', from Detroit, which had been refused admission into the Peninsular Journal, on account of its personal allusions to Dr. N. S. Davis, in which that gentleman, in our estimation, was treated with undeserved unkindness, the editor of our Buffalo contemporary intimates that a bloody contest is still going on between us and the originator of the American Medical Association. As that contest, which was originally confined to the representatives of the rival schools, Professor Palmer and Davis, was sometime since terminated by the assumption of a sort of armed neutrality on the part of the journals through which they had spoken, we think the recent allusion of the Buffalo Journal to the subject is a mere manœuvre, on the part of the editor, by which he may retreat from the field himself, under cover of the smoke from our batteries, which had already ceased firing."

The "communication signed 'R.'" was sent to us with the statement that it had been rejected by the "Peninsular." We cannot say that that important fact had any influence whatever upon our reception of it. We wrote to its author certain inquiries relative to it, and learned that he had no personal acquaintance with Prof. Davis, and that he disclaimed any personal motives. The article itself was moderate in tone, gentlemanly in bearing, and sound in argument. It did not, however, possess that virtue so much prized by our more timid cotemporary, of not meaning anything in particular. It was a *positive* article, and very likely to cause comment.

As to Prof. Davis himself, every one who knows us, knows also that we have a sincere and cordial respect for that gentleman. He holds a high position which he has earned by his own unaided efforts, and we honor him for it. And since the "Peninsular" has thought best to state that it had once rejected the article signed "R.", why did it not tell the whole story and say that its rejection was *conditional?*

As to the contest between the "Northwestern" and the "Peninsular," it had *not* subsided into an "armed neutrality" at the time our sketch was written.

If the editor of the "Peninsular" "thinks that the recent allusion of the Buffalo Journal to the subject is a mere manœuvre on the part of the editor, by which he may retreat from the field himself, under cover of the smoke from our batteries, which had already ceased firing," we trust that the March number of the Buffalo Journal, and its article on the University of Michigan, may correct all such unwarranted suppositions. In that hope we shall say nothing more at this time — except to express our wonder at the very pleasant manner in which the "Peninsular" is soothing its quondam antagonist, the "Northwestern." All through the stormy fall and winter months the "Peninsular" has showered down epithets on the "Northwestern," implying every sin in the Decalogue (though dwelling particularly on the *eighth* commandment) but now the *pleasant May is coming*, and the "Peninsular" is billing and cooing towards the Northwest like any turtle dove!

What does it mean?

